# High YKL-40 Serum Concentration Is Correlated with Prognosis of Chinese Patients with Breast Cancer

**DOI:** 10.1371/journal.pone.0051127

**Published:** 2012-12-05

**Authors:** Dong Wang, Bo Zhai, Fengli Hu, Chang Liu, Jinpeng Zhao, Jun Xu

**Affiliations:** 1 Department of General Surgery, the Fourth Hospital of Harbin Medical University, Harbin, China; 2 Department of Gastroenterology, the Fourth Hospital of Harbin Medical University, Harbin, China; Kinghorn Cancer Centre, Garvan Institute of Medical Research, Australia

## Abstract

This study aimed to investigate the association between serum YKL-40 and prognosis of breast cancer in a Chinese population. Expression of YKL-40 of 120 Chinese patients with breast cancer and 30 controls (benign breast lesions) was measured in tumor tissue by immunohistochemistry and in serum by ELISA. Differences in YKL-40 positivity grouped by specific patients’ characteristics were compared using Pearson Chi-square test for rates of intratumoral staining, one-way ANOVA with a Bonferroni post-hoc comparison, or two-sample t-test for mean YKL-40 serum concentrations. Factors associated with overall survival were identified by univariate and multivariate cox-regression analyses. YKL-40 was elevated in approximately 75% of Chinese patients with breast cancer. A significantly higher percentage of patients with YKL-40 positive tumors had larger tumor size, higher TNM stage, and/or lymph node metastasis. Significantly higher mean YKL-40 serum concentrations were observed in patient subgroups with invasive lobular carcinoma (*P*<0.0167), higher TNM stage (*P*<0.001), and positive lymph node metastasis (*P*<0.001). The estimated mean survival time of patients with YKL-40 positive tumors was significantly shorter than for patients with YKL-40 negative tumors (55.13 months vs 65.78 months, *P* = 0.017). Multivariable Cox-regression analysis identified a significant association of overall survival time with YKL-40 serum concentration. Patients with YKL-40 positive tumors had significantly shorter disease free survival times than those with YKL-40 negative tumors. We propose that the potential utility of YKL-40 intratumoral staining or serum concentration as a biomarker for breast cancer is greatest within 5 years of diagnosis.

## Introduction

Breast cancer is the most frequently diagnosed cancer in women and globally is the leading cause of cancer death in females [1], claiming 458,000 lives worldwide in 2008 [1]. Although most patients with early stage breast cancer are treated with standard therapy (surgery, chemotherapy, and/or radiation) and have a 5 year survival rate of 95%, biomarkers that can accurately predict relapse would be useful for monitoring patients most at-risk for recurrence.

One promising biomarker for predicting risk breast cancer relapse is YKL-40, also called human cartilage glycoprotein-39 (HC gp-39) and chitinase 3-like 1. YKL-40 is expressed in tumor tissue and serum [2], [3]. Treatment with anti-YKL-40 monoclonal antibody inhibited tube formation of microvascular endothelial cells in vitro and inhibited tumor growth, angiogenesis, and progression of brain tumors from U87 cell line in vivo [4].

YKL-40 is a member of family 18 glycosyl hydrolases, although no enzymatic activity has been detected. The YKL-40 gene consists of 10 exons located within 8 kb of DNA on human chromosome 1q32.1, and encodes a protein of 383 amino acids with an N terminal sequence of Tyr-Lys-Leu (YKL), hence the name, YKL-40 [5]. In a Danish population, serum YKL-40 levels ranged from 14–155 mcg/L and this value gradually increased with age (1.5 mcg/L/yr) [6]. YKL-40 plays a role in inflammation, remodeling of extracellular matrix, regulation of cell proliferation, stimulation of angiogenesis [7], and protection against apoptosis [7], [8].

High YKL-40 expression is associated with high grade ovarian cancer [9], and with poor prognosis of endometrial cancer [10], small cell lung cancer [11], glioma [12], colorectal cancer [13], hepatocellular carcinoma [14], gastric cancer [15], and breast cancer [2], [3]. In addition, YKL-40 serum concentrations were higher in women with precancerous lesions than healthy controls [16].

Serum YKL-40 is detectable by ELISA in 19% of non-metastatic breast cancer patients [3] and 30% of metastatic breast cancer patients [17]. Several independent studies demonstrated that high YKL-40 serum concentrations were associated with poor prognosis of breast cancer patients. High YKL-40 serum concentrations were significantly associated with visceral metastases, shorter overall survival [18] and recurrence-free survival [3], as well as aggressiveness of metastases [17]. A high serum YKL-40 level is an independent predictor of overall survival of patients with locally advanced breast cancer [19]. YKL-40 expression in invasive breast cancer cells of patients in the Boston (MA, USA) area was positively correlated with tumor grade, poor differentiation, and expression of HER2/neu [20]. Kim et al [2] showed that high intratumoral expression of YKL-40 in 109 breast tumors from mostly African Americans and Hispanic patients was a significant predictor of post-operative recurrence of breast cancer. However, some studies did not detect a significant association between survival, recurrence-free period, and overall survival. Roslind et al [21] found that intratumoral YKL-40 expression in 630 Danish women with breast cancer was not associated with disease-free survival and overall survival. Factors including types of breast cancer, length of follow-up, types of YKL-40 (secreted versus tumor cell-associated), ethnic origin, sample number, or other factors may account for the disparate results and warrant further studies.

Since the incidence of breast cancer in Asian Americans is less than those of Caucasians and African Americans, but modestly more than those of Hispanic/Latino origins [22], the YKL-40 expression levels may differ among the distinct peoples and breast cancer patients. We hypothesize that ethnic origins or race may affect the utility of YKL-40 as a biomarker for breast cancer. The utility of YKL-40 expression levels as a biomarker of breast cancer in Asians has not been reported. In the present study, the correlation of serum YKL-40 concentrations and intratumoral YKL-40 protein expression in cancer tissues withdisease prognosis was evaluated in breast cancer patients of Chinese origin.

## Materials and Methods

### Patients’ Information

Women with breast cancer were consecutively recruited at the Fourth Hospital of Harbin Medical University from January of 2005 to January of 2009. Before being enrolled in the study, routine chest X-ray, breast mammography and abdominal ultrasonography were performed, but not chemotherapy or radiotherapy. Criteria for exclusion from the study were as follows: bilateral breast cancer or inflammatory breast cancer; metastasis; pre-existing treatment or recurrence of the disease; the presence of diseases that cause an increase in plasma YKL-40 such as liver disease, arthritis, or other cancers. All patients received radical mastectomy or modified radical mastectomy. In the control group, 30 patients with benign breast lesions were recruited. All participants were of Han Chinese origin.

After surgery, patients were followed up every 3 months for 3 years and thereafter every 6 months until January 2012. The protocol was approved by the Ethics Committee of the Fourth Hospital of Harbin Medical University, and informed consent was obtained from each patient before the study. The informed consent was verbal since this study did not change participants’ treatment strategy or health. Doctors recorded the obtainment of the verbal consent in patients’ clinical files. After the surgery agreement was signed but prior to the operation, the patients also noted in the surgical agreement that he/she was informed about and agreed to participate in this study. The Ethics Committee approved this verbal consent procedure and had unscheduled inspection of documents and records to assure the study was compliant.

### Detection of YKL-40 Expression in Breast Cancer Tissue by Immunohistochemistry

The YKL-40 protein was detected by immunohistochemistry using PV9000 kit for immunohistochemistry and DAB (Beijing Zhongshan Golden Bridge Biotech Co., Ltd) which is a streptavidin peroxidase based detection method. In brief, paraffin was removed 3 µm tissues sections with xylene. After incubation in 10 mmol/L pH 6.0 citrate buffer, antigen was retrieved heating in a microwave oven. The sections were washed with flowing water and allowed to cool to room temperature (r.t.). After inactivating the endogenous peroxidase (0.3% H_2_O_2,_ r.t., 10 min), sections were incubated with YKL-40 antibody (1∶200; rabbit anti-human YKL-40 monoclonal antibody, QUIDEL, USA) at 4°C overnight. After washing 3 times in PBS (5 min each), sections were incubated with biotin labeled secondary antibody (mouse anti-rabbit IgG) at r.t. for 30 min, washed 3 times in PBS and visualized by incubating at r.t. for 15 min followed by counterstaining with haematoxylin for 15 min. In the negative control group, the primary antibody was replaced with PBS.

**Figure 1:**
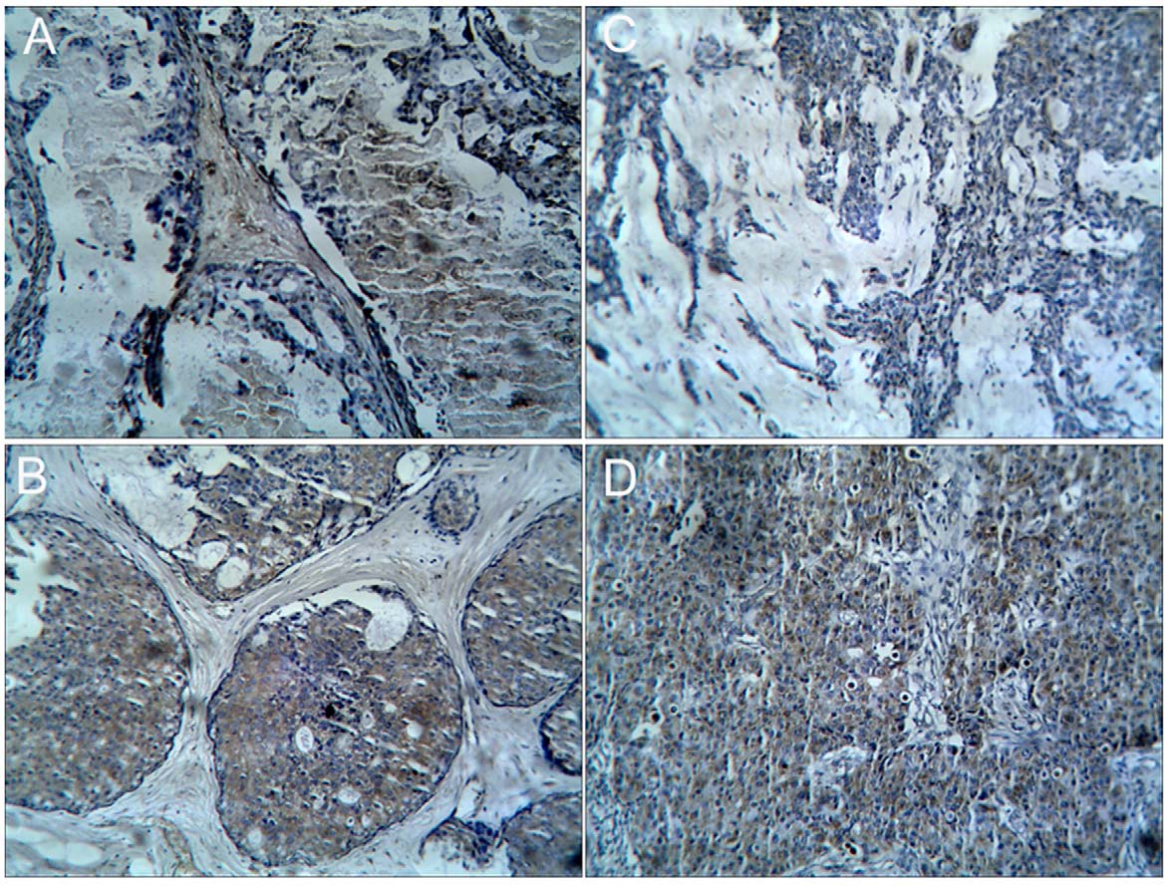
Representative photographs of YKL-40 immunohistochemical staining for positive cell and intensity scoring. A) Noninvasive ductal carcinoma staining at positive score 1 and intensity score 2, B) Noninvasive ductal carcinoma staining at positive score 3 and intensity score 2. C) Invasive ductal carcinoma staining positive score 2 and intensity score 2. D) Invasive ductal carcinoma staining positive score 3 and intensity score 2. All photos had x100 magnification.

The previously described semi-quantitative method was used to score tumor samples for extent and intensity of YKL-40 staining [21]. The number of YKL-40 positive cells was determined and then scored as follows: 0, negative; 1, ≤10% positive cells; 2, 11%–50% positive cells; 3, ≥51% positive cells. Staining intensity was scored as follows: 0, no staining; 1, light yellow; 2, yellow-brown; 3, brown. Representative staining of positive cells and intensity scoring are shown in Figure 1. Scores were added and a composite YKL-40 staining score of ≥3 was defined as positive immunoreactivity, as previously described [21].

### Detection of Serum YKL-40 by ELISA

One day before surgery, venous blood (3∼5 ml) was obtained, clotted, and centrifuged at 3000 r/min for 10 min. Serum was collected, divided into aliquots, and stored at −80°C. Concentrations of YKL-40 in serum were measured in the YKL-40 ELISA kit (Shanghai Lanji Biotech Co., Ltd), according to manufacturer’s instructions. In brief, 50 µl of standard (dilution: 1∶5) or samples was added to corresponding wells and plates were incubated for 30 min at 37°C. After washing, 50 µl of labeling reagent was added to each well and the plate incubated for 30 min at 37°C. For visualization of the reactions, after washing, plates were incubated at 37°C in dark for 10 min. After addition of stop solution, absorbance was measured at 450 nm on a Biotek-elx800 microplate reader (Roche, USA), and the YKL-40 concentration determined.

### Statistical Analysis

The study included 120 cases and 30 control subjects. Based on the observed YKL-40 serum levels for case and controls (72.6±27.2 µg/L vs. 36.8±12.9 µg/L, respectively), the power was calculated to be almost 100% for identifying a difference in YKL-40 serum levels between the 2 groups. The probability of a type 1 error probability for the null hypothesis was 0.05 (α = 0.05).

The key outcomes measured in this study were overall survival and disease-free survival. Other outcomes included age, tumor size, tumor type, TNM stage, presence or absence of lymph node metastasis, estrogen receptor (ER) and progesterone receptor (PR). In this study, tumor types included noninvasive ductal carcinoma, invasive lobular carcinoma, and invasive ductal carcinoma.

Rates of YKL-40 intratumoral staining were summarized as n (%) and serum YKL-40 level as mean ± standard deviations (SD) for case and control group, respectively. Positive rates of intratumoral staining were grouped by specific patients’ characteristics and compared by using Pearson Chi-square test. Differences in serum YKL-40 levels between specific patient characteristics were compared by using one-way ANOVA with a Bonferroni post-hoc comparison or two-sample t-test. Survival times between groups were displayed by Kaplan-Meier curves after a log-rank test. Univariable and multivariable cox-regression models were performed to identify factors associated with the overall survival time. Variables with significant P-values <0.05 in univariable cox-regression model were selected and analyzed by a multivatirate cox-regression model. All statistical assessments were considered significant at P-value <0.05. An adjusted significance level 0.0167 (0.05/3) was also considered for the Bonferroni adjustment approach. Statistical analyses were performed by using PASW 18.0 statistics software (SPSS Inc, Chicago, IL, USA).

## Results

### Demographics of Patients and Controls

The average ages of the 120 breast cancer patients were similar to those of the 30 controls (48.4±12.4 yrs vs 46.7±12.4 yrs, *P* = 0.056). The frequency of expression of the estrogen receptor and progesterone receptors were significantly different between the breast cancer patients and controls (data not shown). The intensity of YKL-40 staining and the percentage of YKL-40 positive cells were scored (Figure 1) and their sum formed a composite scale for comparing YKL-40 staining among samples and groups. Representative positive YKL-40 staining in the 3 pathological types of breast cancer (noninvasive ductal carcinoma [n = 36], invasive lobular carcinoma [n = 24], and invasive ductal carcinoma [n = 60]) are shown in supplemental Figures S1B, S1C, and S1D, respectively.

### Association of YKL-40 Intratumoral Expression and YKL-40 Serum Concentrations

**Figure 2:**
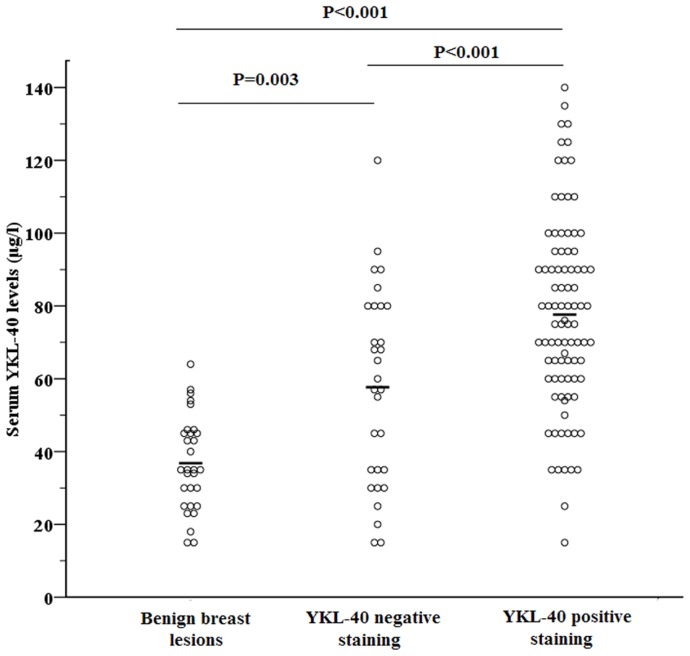
Individual concentrations of serum YKL-40 in the controls (n = 30), and breast cancer patients who were YKL-40 negative (n = 30) or YKL-40 positive by IHC (n = 90). Bold lines indicate the mean of each subgroup. (control: 36.8±12.9 µg/l; YKL-40 negative staining: 56.7±26.9 µg/l; YKL-40 positive staining: 77.6±26.3 µg/l). P-value was derived from one-way ANOVA with a post-hoc Bonferroni comparison for comparing the difference in YKL-40 concentration among groups.

The breast cancer group had a significantly higher rate of positive YKL-40 intratumoral staining than the control group (75% [90/120] vs 20% [6/30], *P*<0.001). The breast cancer group also had a higher mean serum concentration of YKL-40 (72.6±27.7 g/L) than the controls (36.8±12.9 µg/L; *P*<0.001). The YKL-40 serum concentrations varied widely within each of the 3 groups (i.e., controls, subgroup of breast cancer patients with YKL-40 positive tumors, and subgroup of breast cancer patients with YKL-40 negative or poorly stained tumors) (Figure 2). The mean serum levels of YKL-40 controls (36.8±12.9 µg/L) was significantly lower than that of patients in the YKL-40 negative tumor group (56.7±26.9 µg/L, *P* = 0.003). The YKL-40 negative tumor groups had was significantly lower levels than patients with positive YKL-40 stained tumors (77.6±26.3 µg/L, *P*<0.001) (Figure 2).

Breast cancer patients were grouped by their YKL-40 IHC staining score to assess a potential relationship between stainining and YKL-40 serum levels (Table S1). The YKL-40 IHC staining score significantly correlated with the mean YKL-40 serum concentration for each breast cancer subgroup (r = 0.294, *P* = 0.001).

### Association of YKL-40 Intratumoral Expression with Tumor Characteristics

To explore YKL-40’s potential role as a prognostic biomarker of breast cancer patients, we next analyzed the associations of patients’ characteristics with mean serum concentrations in the patients stratified by the status of YKL-40 intratumoral staining (Table 1). Patients with tumor size >2 cm, TNM stage II or III, and positive lymph node metastasis had a higher rate of YKL-40 positive tumors (all *P*-values <0.05). Invasive lobular carcinoma of the YKL-40-positive tumor group, but not the YKL-40 negative tumor group, had significantly higher YKL-40 serum concentrations. Differences in the mean YKL-40 concentrations of patients with invasive ductal carcinoma and noninvasive ductal carcinoma were not significant between the two subgroups of breast cancer patients. In the YKL-40-positive tumor compared with the YKL-40 negative subgroup of cancer patients, significantly higher mean serum YKL-40 levels were observed in patients with invasive lobular carcinoma (*P*<0.0167), TNM stage II or III (*P*<0.001), positive lymph node metastasis (*P* = 0.008), or who were deceased (*P* = 0.034). Lower serum levels of YKL-40, in the YKL-40 negative tumor group, but not the YKL-40 positive tumor group, were associated with middle age (*P* = 0.02). Patients in the YKL-40 negative tumor group, but not in the YKL-40 positive group, who expressed the progesterone receptor had higher levels of serum YKL-40 (*P*<0.05). The status of the estrogen receptor did not correlate with the mean YKL-40 serum concentrations in either breast cancer subgroup. Patients who died had significantly higher mean YKL-40 serum concentrations compared with those that did not.

**Table 1:** Association of patients’ characteristics with rate of positive YKL-40 intratumoral staining and YKL-40 serum levels.

	YKL-40 intratumoral staining (n = 120)			Serum YKL-40 levels (positive YKL-40 tumor group, (n = 90)		Serum YKL-40 levels (negative YKL-40 tumor group, (n = 30)	
Characteristics	Negative	Positive	P-value	Mean±SD	P-value	Mean±SD	P-value
Age			0.440		0.199		0.020*
≦40 years	14 (30.4%)	32 (69.6%)		72.38±26.55		59.29±28.87	
40 to 60 years	12 (24.0%)	38 (76.0%)		83.34±29.54		45.83±20.76	
≧60 years	4 (16.7%)	20 (83.3%)		75.20±16.57		87.50±6.45^b^	
Tumor size, cm			0.001*		0.631		0.405
≦2 cm	22 (39.3%)	34 (60.7%)		75.91±28.96		60.18±28.76	
>2 cm	8 (12.5%)	56 (87.5%)		78.68±27.78		50.75±7.43	
Pathological classification			0.052		0.001*		0.141
Noninvasive ductal carcinoma	10 (27.8%)	26 (72.2%)		66.35±19.32		62.50±27.00	
Invasive lobular carcinoma	10 (41.7%)	14 (58.3%)		98.43±22.28^a^		66.40±26.37	
Invasive ductal carcinoma	10 (16.7%)	50 (83.3%)		77.68±27.28		44.10±24.32	
TNM staging			0.006*		<0.001*		0.560
I	14 (46.7%)	16 (53.3%)		55.31±17.93		52.43±25.22	
II	12 (19.4%)	50 (80.6%)		77.16**±**23.39^a^		60.42±32.37	
III	4 (14.3%)	24 (85.7%)		93.50±26.34^ab^		67.75±10.21	
Lymph node metastasis			0.003*		0.008*		0.287
Negative	20 (38.5%)	32 (61.5%)		67.88±25.36		53.45±23.88	
Positive	10 (14.7%)	58 (85.3%)		83.02±25.46		66.10±31.77	
Estrogen Receptor			0.507		0.913		0.056
Negative	12 (28.6%)	30 (71.4%)		78.07±27.72		46.25±22.57	
Positive	18 (23.1%)	60 (76.9%)		77.42±25.81		65.28±27.41	
Progesterone Receptor			0.398		0.627		0.020*
Negative	16 (28.6%)	40 (71.4%)		79.15±26.60		47.19±21.90	
Positive	14 (21.9%)	50 (78.1%)		76.42±26.29		69.64±27.77	
Survival status			0.292		0.034*		0.010*
Deceased	4 (16.7%)	20 (83.3%)		91.70±33.83		88.75±23.23	
Alive	26 (27.1%)	70 (72.9%)		73.61±22.45		52.88±24.42	

YKL-40 intratumoral staining of each breast cancer patient were compiled as n (%) and differences in YKL-40 intratumoral staining between specific patients’ characteristics were compared using Pearson Chi-square test.

YKL-40 serum concentrations for each characteristic were presented as mean±SD. Differences in serum YKL-40 levels between specific patients’ characteristics were compared by using one-way ANOVA with post-hoc Bonferroni comparison or two-sample t-test.

* P<0.05, indicated a significance.

a,b P<0.0167 (0.05/3), indicated a significant difference compared with the ^a^first category and ^b^second category of the specific variable.

### YKL-40 Expression Associated with Shorter Overall Survival and DFS

To further assess the association of YKL-40 expression with survival, survival times and tumor characteristics were analyzed by univariate and multivariate Cox-regression models (Table 1). Univariate Cox-regression analysis indicated that shorter survival time was associated with positive YKL-40 intratumor expression, YKL-40 serum levels, tumor size, invasive ductal carcinoma, TNM stage, and presence of lymph node metastasis. Patients with YKL-40-positive tumors, higher YKL-40 serum concentrations, larger tumor size, invasive ductal carcinoma, TNM stage III, or positive lymph node metastasis had a higher risk for shorter survival time. However, in the multivariate Cox-regression analysis, serum YKL-40 concentration was the only variable associated with overall survival (Table 2).

**Table 2:** Univariate and multivariate cox-regression model for overall survival time analysis.

	Univariate		Multivariate	
Variables	HR (95%CI.)	P-value	HR (95%CI.)	P-value
YKL-40 intratumoral staining				
Negative	Reference		Reference	
Positive	3.46 (1.15–10.39)	0.027	1.60 (0.40–6.34)	0.504
YKL-40 serum level	1.05 (1.03–1.07)	<0.001*	1.04 (1.02–1.06)	<0.001*
Age				
≦40 years	Reference		NA	
40 to 60 years	1.80 (0.73–4.43)	0.203		
≧60 years	2.02 (0.59–6.89)	0.261		
Tumor size, cm				
≦2 cm	Reference			
>2 cm	3.79 (1.52–9.44)	0.004*	3.74 (0.71–19.68)	0.120
Pathological classification				
noninvasive ductal carcinoma	Reference		Reference	
Invasive lobular carcinoma	2.25 (0.75–6.73)	0.147	1.99 (0.41–9.65)	0.391
invasive ductal carcinoma	3.64 (1.26–10.51)	0.017*	2.07 (0.43–9.87)	0.363
TNM stage				
I	Reference			
II	1.99 (0.62–6.45)	0.249	0 (0–3.08×10^96^)	0.923
III	16.45 (4.32–62.62)	<0.001*	0 (0–4.88×10^96^)	0.926
Lymph node metastasis				
Negative	Reference		Reference	
Positive	5.08 (1.72–14.95)	0.003*	0.6×10^6^ (0–1.92×10^6^)	0.926
ER				
Negative	Reference		NA	
Positive	0.65 (0.27–1.54)	0.324		
PR				
Negative	Reference		NA	
Positive	1.01 (0.43–2.40)	0.981		

Results were presents as estimated Hazard Ratio with 95% confidence interval (HR (95%CI.)) for specific variables.

Variables with a significant P-value (<0.05) in univariate cox-regression model were selected and analyzed by using multivariate cox-regression model.

NA, not assessed.

* P<0.05, indicated a significant association.

Factors associated with duration of disease-free survival were also analyzed by univariate and multivariate Cox regression analysis (Table S2). Univariate analysis indicated that YKL-40 intratumoral expression, YKL-40 serum concentrations, tumor size >2 cm, invasive lobular and ductal carcinomas, TNM stage, and ER and PR expression were significantly associated with shorter disease-free survival. Multivariate analysis indicated that YKL-40 intratumoral expression, YKL-40 serum concentrations, invasive lobular and ductal carcinomas, and progesterone receptor expression were associated with shorter disease-free survival (Table S2).

**Figure 3:**
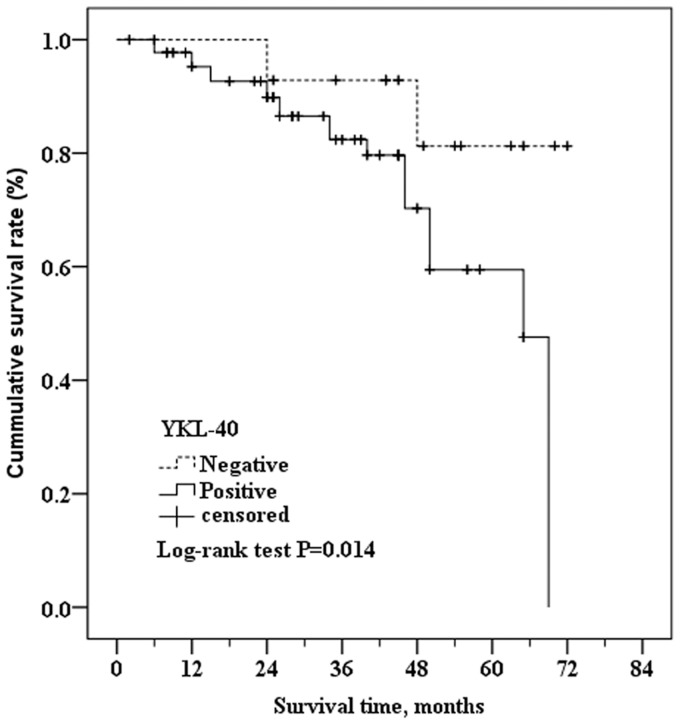
Kaplan-Meier survival curves of breast cancer patients with positive YKL-40 intratumoral staining versus those with negative YKL-40 intratumoral staining. The Log-rank test showed that the survival times were significantly shorter in patients with positive YKL-40 intratumoral staining (55.13 months.95%CI.: 49.69 to 60.58 months) than those with negative YKL-40 intratumoral staining (65.78 months, 95%CI: 60.17 to 71.40 months) (P = 0.014).

**Figure 4:**
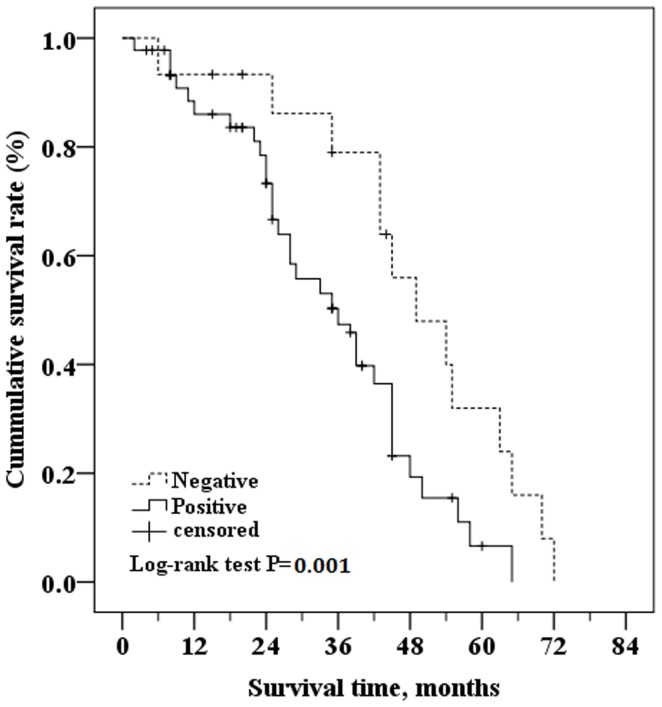
Kaplan-Meier curve comparing disease free survival of patients with positive YKL-40 intratumoral staining versus those with negative YKL-40 intratumoral staining. Log-rank test determined that the disease-free survival in YKL-40 positive tumor group was 36 months (95%CI.: 28.95 to 43.05 months)), which was significantly shorter than those in the YKL-40 negative tumor group (49 months (95%CI: 38.23 to 59.77 months) (P = 0.001).

Kaplan-Meier curves of patients with YKL-40 positive and negative tumor tissue indicated that there was a significant difference in survival times between groups (Figure 3). The estimated mean survival time of patients with positive YKL-40 intratumoral expression was 55.13 months (95% CI: 49.69 to 60.58 months) whereas that of patients with negative tumor tissue was significantly longer at 65.78 months (95% CI: 60.17 to 71.40 months; *P* = 0.017 as determined by the Log-rank test) (Figure 3). Similarly, the Kaplan-Meier analysis and the Log-rank test showed that the disease-free survival time was significantly shorter in patients with YKL-40 positive intratumoral staining (36 months [95% CI: 28.95 to 43.05 months]) than those with YKL-40 negative intratumoral staining (49 months [95% CI: 38.23 to 59.77 months]; *P* = 0.001) (Figure 4).

**Figure 5:**
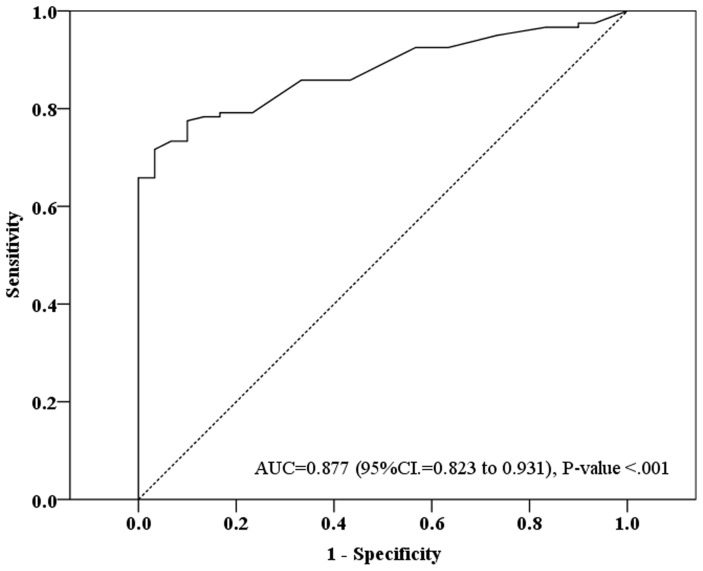
ROC curve of the serum YKL-40 levels of 120 breast cancer patients and 30 controls. The estimated area under the ROC curve was observed as AUC = 0.877 with 95%CI. = 0.823 to 0.931 (P-value<.001) The best cut-off of serum YKL-40 level was observed as “60” based on the maximization of Yuden index. The predictive diagnosis of breast cancer according the cut-off of serum level = 60 was 65.8% in sensitivity, 96.7% in specificity, 98.8% in PPV, and 41.4% in NPV.

ROC curves were calculated based on the YKL-40 serum levels of the 120 breast cancer patients and 30 controls (Figure 5). The estimated area under the ROC curve (AUC) was 0.877 (95% CI: 0.823 to 0.93; *P*-value<0.001) The opptimal cut-off of serum YKL-40 level was 60 based on the maximization of Yuden index, resulting in. 65.8% sensitivity, 96.7% specificity, 98.8% positive predictive value (PPV), and 41.4% negative predictive value (NPV).

## Discussion

The utility of YKL-40 expression in the serum or breast cancer tissue as a biomarker of disease-free survival or overall survival is promising but controversial [2], [17], [18], [20], [21], [23]. Here we investigated whether YKL-40 serum concentration or its intratumoral expression correlated with characteristics of breast cancer or prognosis (disease-free survival, overall survival) in Chinese patients. Although 30 of 120 tumors were negative for YKL-40 expression in this study, the prevalence of YKL-40 tumor cell expression was significantly higher in more advanced tumors, similar to Shao et al [20] but not Kim et al [2]. In our study, high YKL-40 intratumoral expression was also significantly more prevalent breast cancer patients with metastases compared with patients who did not have metastatic disease, which agrees with the studies of Johansen et al, and Jensen et al. [17], [18]. In both our and Shao et al.’s studies [20], the intratumoral YKL-40 expression did not significantly correlate with histopathological type. Although the percent of patients with YKL-40 positive tumors was not significantly associated with death (Table 2), log rank analysis showed that shorter survival times were significantly associated with YKL-40 positive tumors, similar to the investigations of Kim et al and Jensen et al [2], [17], but in disagreement with the findings of Roslind et al [21].

YKL-40 serum concentrations may provide a more consistent biomarker of a specific patient disease progression as intratumoral YKL-40 expression can vary across a single breast cancer nodule [21]. High YKL-40 serum concentrations were significantly associated with invasive lobular carcinoma, TMN stage III, lymph node metastases, and death in our study. Similarly, Yamac et al found high YKL-40 serum concentrations were associated with early death [19]. A significant association was observed between metastases-positive breast cancer and high YKL-40 serum concentrations in several studies [17], [18] including ours but not in node-positive breast cancer in the study of Yamac et al [19]. Differences between correlation results in this study and others may represent differences in populations and study design.

To further confirm the correlation between YKL-40 and disease prognosis, Cox proportional risk model was employed to evaluate survival as a function of YKL-40 expression. The patients who had tumors that did not expressed YKL-40 had longer estimated mean overall survival (65.78 months) than those with YKL-40 positive tumor. These findings are consistent with results in breast cancer patients of Black American and Hispanic origins [2]. However, Roslind et al [21] found no significant association between survival and YKL-40 tumor expression in a study of 630 breast cancer patients from Denmark. Most of the 630 patients had YKL-40 positive tumors (98%). Their mean overall survival time was 14 years and 11 months, and mortality was 48% [21]. The median disease-free survival was as long as 7 years and 10 months [21]. The duration of their disease matched the follow-up period in the present study.

One possible interpretation is that the association between YKL-40 intratumoral expression and DFS may be influenced by the duration of the study. As shown in our study, at early and intermediate stages of follow-up, the YKL-40 positive tumor group had a shorter overall survival and disease-free survival than the YKL-40 negative tumor group. However, at the end of follow-up, the difference in survival rate between the two groups had narrowed. The study by Kim et al had a relatively short median follow-up period (3.2 years), and a low percentage of patients with YKL-40 positive tumors (34%), but the survival analysis showed that positive YKL-40 expression was an independent predictor of early recurrence. Thus, we speculate that YKL-40 expression is associated with breast cancer prognosis. In breast cancer patients, positive YKL-40 expression was associated with poorer prognosis following surgery. With a longer follow-up period, there is a lower correlation between YKL-40 expression and prognosis. Confirmation of this hypothesis and elucidation of the specific mechanism requires further studies.

Tumor stage, size, the presence of invasive ductal carcinoma, or lymph node metastasis correlated with survival when assayed by univariate analysis but not when evaluated by multivariate analysis. This was not anticipated. It is possible that the lack of correlation of these factors with survival by multivariate analysis may be due to the small sample size, short-term follow-up, and the small number of deaths during the study. Although not evaluated, it is possible that YKL-40 serum levels may correlate with these variables.

Limitations of this study include small sample size and larger sample sizes are required to further test the correlation between YKL-40 expression and prognosis. In addition, an increase in YKL-40 serum levels is a non-specific marker because YKL-40 serum concentrations can be increased in patients with several unrelated inflammatory diseases. Thus, if the serum YKL-40 expression is used as a marker of breast cancer, it is necessary to exclude false positive samples. Our study focused on the levels of YKL-40 in breast cancer patients of Chinese origin and the findings may not be applicable to all ethnic groups [21].

### Conclusions

YKL-40 is a novel marker of breast cancer that is elevated in approximately 75% of Chinese patients and higher YKL-40 expression was significantly associated with a poorer prognosis of breast cancer over 7-year follow-up. Understanding the molecular mechanisms that underlie the correlation between the YKL-40 expression and breast cancer outcomes may provide novel targets for therapy.

## Supporting Information

Figure S1 **YKL-40 staining in different pathological types of breast cancer (A) noninvasive ductal carcinoma, negative YKL-40 staining (x100), (B) noninvasive ductal carcinoma, positive YKL-40 staining (x400), (C) positive YKL-40 stained invasive lobular carcinoma, (x100) and (D) positive YKL-40 stained invasive ductal carcinoma (x400).**
(TIF)

Table S1 **YKL-40 IHC staining score and mean serum levels of YKL-40 in breast cancer patients.**
(DOC)

Table S2 **Cox-regression analysis of Disease Free Survival (DFS).**
(DOC)

## References

[1] Jemal A, Bray F (2011) Center MM, Ferlay J, Ward E, et al (2011) Global cancer statistics. CA Cancer J Clin 61(2): 69–90. 21296855 10.3322/caac.20107

[2] Kim SH, Das K, Noreen S, Coffman F, Hameed M (2007) Prognostic implications of immunohistochemically detected YKL-40 expression in breast cancer. World J Surg Oncol 5: 17. 17286869 10.1186/1477-7819-5-17 PMC1802867

[3] Johansen JS, Christensen IJ, Riisbro R, Greenall M, Han C, et al (2003) High serum YKL-40 levels in patients with primary breast cancer is related to short recurrence free survival. Breast Cancer Res Treat 80(1): 15–21. 12889595 10.1023/A:1024431000710

[4] Faibish M, Francescone R, Bentley B, Yan W, Shao R (2011) A YKL-40-neutralizing antibody blocks tumor angiogenesis and progression: a potential therapeutic agent in cancers. Mol Cancer Ther 10(5): 742–751. 21357475 10.1158/1535-7163.MCT-10-0868 PMC3091949

[5] Shackelton LM, Mann DM, Millis AJ (1995) Identification of a 38-kDa heparin-binding glycoprotein (gp38k) in differentiating vascular smooth muscle cells as a member of a group of proteins associated with tissue remodeling. J Biol Chem 270(22): 13076–13083. 7768902 10.1074/jbc.270.22.13076

[6] Bojesen SE, Johansen JS, Nordestgaard BG (2011) Plasma YKL-40 levels in healthy subjects from the general population. Clin Chim Acta 412(9–10): 709–712. 21272568 10.1016/j.cca.2011.01.022

[7] Shao R, Hamel K, Petersen L, Cao QJ, Arenas RB, et al (2009) YKL-40, a secreted glycoprotein, promotes tumor angiogenesis. Oncogene 28(50): 4456–4468. 19767768 10.1038/onc.2009.292 PMC2795793

[8] Junker N, Johansen JS, Andersen CB, Kristjansen PE (2005) Expression of YKL-40 by peritumoral macrophages in human small cell lung cancer. Lung Cancer 48(2): 223–231. 15829322 10.1016/j.lungcan.2004.11.011

[9] Stawerski P, Wagrowska-Danilewicz M, Stasikowska-Kanicka O, Danilewicz M (2011) Increased tissue immunoexpression of YKL-40 protein in high grade serous ovarian cancers. Pathol Res Pract 207(9): 573–576. 21820815 10.1016/j.prp.2011.06.008

[10] Peng C, Peng J, Jiang L, You Q, Zheng J, et al (2010) YKL-40 protein levels and clinical outcome of human endometrial cancer. J Int Med Res 38(4): 1448–1457. 20926018 10.1177/147323001003800427

[11] Thom I, Andritzky B, Schuch G, Burkholder I, Edler L, et al (2010) Elevated pretreatment serum concentration of YKL-40-An independent prognostic biomarker for poor survival in patients with metastatic nonsmall cell lung cancer. Cancer 116(17): 4114–4121. 20564116 10.1002/cncr.25196

[12] Francescone RA, Scully S, Faibish M, Taylor SL, Oh D, et al (2011) Role of YKL-40 in the angiogenesis, radioresistance, and progression of glioblastoma. J Biol Chem 286(17): 15332–15343. 21385870 10.1074/jbc.M110.212514 PMC3083166

[13] Cintin C, Johansen JS, Christensen IJ, Price PA, Sorensen S, et al (1999) Serum YKL-40 and colorectal cancer. Br J Cancer 79(9–10): 1494–1499. 10188896 10.1038/sj.bjc.6690238 PMC2362720

[14] Xiao XQ, Hassanein T, Li QF, Liu W, Zheng YH, et al (2011) YKL-40 expression in human hepatocellular carcinoma: a potential biomarker? Hepatobiliary Pancreat Dis Int 10(6): 605–610. 22146624 10.1016/s1499-3872(11)60103-3

[15] Bi J, Lau SH, Lv ZL, Xie D, Li W, et al (2009) Overexpression of YKL-40 is an independent prognostic marker in gastric cancer. Hum Pathol 40(12): 1790–1797. 19765801 10.1016/j.humpath.2009.07.005

[16] Qin W, Zhu W, Schlatter L, Miick R, Loy TS, et al (2007) Increased expression of the inflammatory protein YKL-40 in precancers of the breast. Int J Cancer 121(7): 1536–1542. 17565739 10.1002/ijc.22881

[17] Jensen BV, Johansen JS, Price PA (2003) High levels of serum HER-2/neu and YKL-40 independently reflect aggressiveness of metastatic breast cancer. Clinical cancer research 9(12): 4423–4434. 14555515

[18] Johansen JS, Cintin C, Jorgensen M, Kamby C, Price PA (1995) Serum YKL-40: a new potential marker of prognosis and location of metastases of patients with recurrent breast cancer. Eur J Cancer 31A(9): 1437–1442. 7577068 10.1016/0959-8049(95)00196-p

[19] Yamac D, Ozturk B, Coskun U, Tekin E, Sancak B, et al (2008) Serum YKL-40 levels as a prognostic factor in patients with locally advanced breast cancer. Adv Ther 25(8): 801–809. 18670741 10.1007/s12325-008-0082-2

[20] Shao R, Cao QJ, Arenas RB, Bigelow C, Bentley B, et al (2011) Breast cancer expression of YKL-40 correlates with tumour grade, poor differentiation, and other cancer markers. Br J Cancer 105(8): 1203–1209. 21934681 10.1038/bjc.2011.347 PMC3208489

[21] Roslind A, Knoop AS, Jensen MB, Johansen JS, Nielsen DL, et al (2008) YKL-40 protein expression is not a prognostic marker in patients with primary breast cancer. Breast Cancer Res Treat 112(2): 275–285. 18157633 10.1007/s10549-007-9870-7

[22] American Cancer Society (2011) Breast Cancer Facts and Figures 2011–2012. In. Atlanta: American Cancer Society, Inc.

[23] Johansen JS, Schultz NA, Jensen BV (2009) Plasma YKL-40: a potential new cancer biomarker? Future Oncol 5: 1065–1082. 19792974 10.2217/fon.09.66

