# Supplementary figures and images for: High YKL-40 Serum Concentration Is Correlated with Prognosis of Chinese Patients with Breast Cancer
Source: PLoS One. 2012 Dec 5;7(12):e51127. doi: 10.1371/journal.pone.0051127 (PMC3515550; doi:10.1371/journal.pone.0051127)

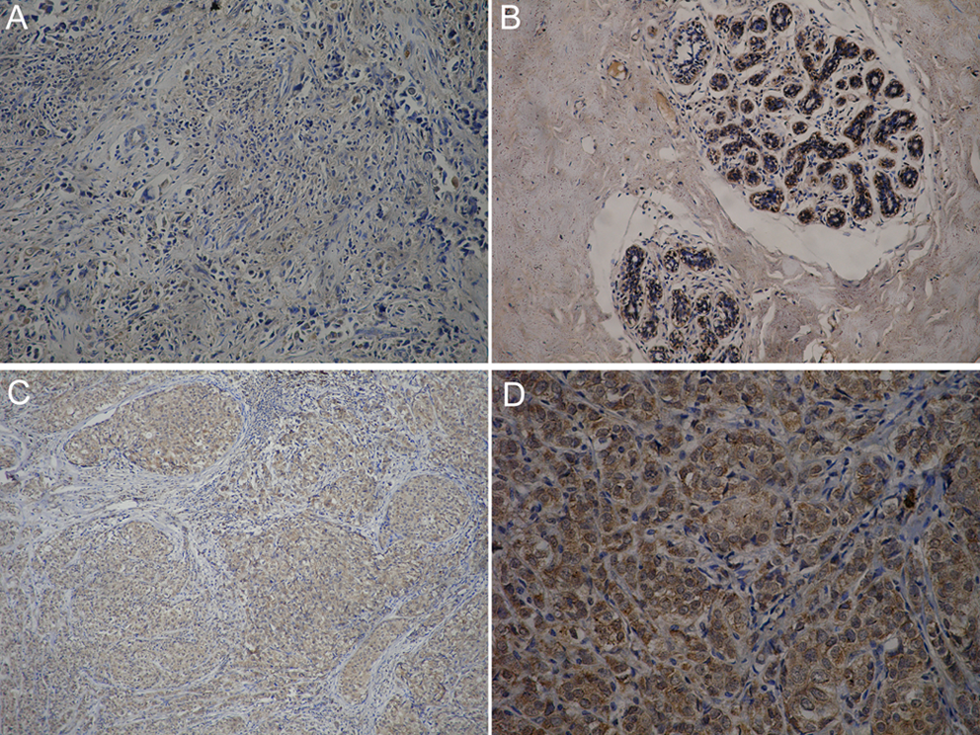

Supplement: Figure S1 — YKL-40 staining in different pathological types of breast cancer (A) noninvasive ductal carcinoma, negative YKL-40 staining (x100), (B) noninvasive ductal carcinoma, positive YKL-40 staining (x400), (C) positive YKL-40 stained invasive lobular carcinoma, (x100) and (D) positive YKL-40 stained invasive ductal carcinoma (x400). (TIF) [file pone.0051127.s001.tif]
