# Supplementary material for: High YKL-40 Serum Concentration Is Correlated with Prognosis of Chinese Patients with Breast Cancer
Source: PLoS One. 2012 Dec 5;7(12):e51127. doi: 10.1371/journal.pone.0051127 (PMC3515550; doi:10.1371/journal.pone.0051127)
Supplement: Table S1 — YKL-40 IHC staining score and mean serum levels of YKL-40 in breast cancer patients. (DOC) [file pone.0051127.s002.doc]

Supplemental table S1. YKL-40 IHC staining score and mean serum levels of YKL-40 in breast cancer patients

| Category | YKL-40  IHC score | N | Serum YKL-40 level  (Mean±SD) |
| --- | --- | --- | --- |
| Negative | 0 | 8 | 62.50±21.37 |
|  | 2 | 22 | 55.91±28.89 |
| Positive | 3 | 30 | 74.67±23.38 |
|  | 4 | 40 | 76.70±29.23 |
|  | 5 | 16 | 80.56±21.90 |
|  | 6 | 4 | 97.50±32.79 |
|  | Total | 120 | 72.64±27.74 |
