# Supplementary material for: High YKL-40 Serum Concentration Is Correlated with Prognosis of Chinese Patients with Breast Cancer
Source: PLoS One. 2012 Dec 5;7(12):e51127. doi: 10.1371/journal.pone.0051127 (PMC3515550; doi:10.1371/journal.pone.0051127)
Supplement: Table S2 — Cox-regression analysis of Disease Free Survival (DFS). (DOC) [file pone.0051127.s003.doc]

Supplemental table S2. Cox-regression analysis of Disease Free Survival (DFS)

|  | Univariate | |  | Multivariate | |
| --- | --- | --- | --- | --- | --- |
| Variables | HR (95%CI.) | P-value |  | HR (95%CI.) | P-value |
| YKL-40 intratumoral staining |  |  |  |  |  |
| Negative | Reference |  |  | Reference |  |
| Positive | 2.24 (1.36 , 3.70) | 0.002^*^ |  | 3.08 (1.59 , 5.99) | 0.001^*^ |
| YKL-40 serum level | 1.01 (1.00 , 1.02) | 0.018^*^ |  | 1.02 (1.00 , 1.03) | 0.008^*^ |
| Age |  |  |  |  |  |
| ≦40 years | Reference |  |  | Reference |  |
| 40 to 60 years | 0.99 (0.61 , 1.60) | 0.970 |  | 0.69 (0.39 , 1.23) | 0.209 |
| ≧60 years | 2.31 (1.32 , 4.04) | 0.003^*^ |  | 1.46 (0.64 , 3.34) | 0.365 |
| Tumor size, cm |  |  |  |  |  |
| ≦2cm | Reference |  |  | Reference |  |
| >2cm | 2.94 (1.88 , 4.59) | <.001^*^ |  | 2.07 (0.82 , 5.26) | 0.125 |
| Pathological classification |  |  |  |  |  |
| Noninvasive ductal carcinoma | Reference |  |  | Reference |  |
| Invasive lobular carcinoma | 2.21 (1.17 , 4.19) | 0.015^*^ |  | 3.75 (1.42 , 9.86) | 0.008^*^ |
| Invasive ductal carcinoma | 6.08 (3.58 , 10.33) | <.001^*^ |  | 6.01 (2.53 , 14.29) | <.001^*^ |
| TNM stage |  |  |  |  |  |
| I | Reference |  |  | Reference |  |
| II | 1.83 (1.11 , 3.01) | 0.018^*^ |  | 0.37 (0.14 , 0.99) | 0.047^*^ |
| III | 5.03 (2.52 , 10.06) | <.001^*^ |  | 0.34 (0.09 , 1.21) | 0.096 |
| Lymph node metastasis |  |  |  |  |  |
| Negative | Reference |  |  | NA |  |
| Positive | 1.21 (0.80 , 1.85) | 0.363 |  |  |  |
| ER | |  |  |  |  |
| Negative | Reference |  |  | Reference |  |
| Positive | 0.43 (0.28 , 0.67) | <.001^*^ |  | 0.69 (0.40 , 1.21) | 0.195 |
| PR | |  |  |  |  |
| Negative | Reference |  |  | Reference |  |
| Positive | 0.49 (0.32 , 0.74) | 0.001^*^ |  | 0.44 (0.24 , 0.80) | 0.007^*^ |
